# Non-replicating Vaccinia Virus TianTan Strain (NTV) Translation Arrest of Viral Late Protein Synthesis Associated With Anti-viral Host Factor SAMD9

**DOI:** 10.3389/fcimb.2020.00116

**Published:** 2020-03-20

**Authors:** Ying Zhao, Li Zhao, Panpan Huang, Jiao Ren, Peng Zhang, Houwen Tian, Wenjie Tan

**Affiliations:** ^1^NHC Key Laboratory of Medical Virology and Viral Disease, Chinese Center for Disease Control and Prevention, National Institute for Viral Disease Control and Prevention, Beijing, China; ^2^Shenzhen Research Institute, City University of Hong Kong, Shenzhen, China

**Keywords:** non-replicating vaccinia virus TianTan strain, viral vector, replication inhibition, SAMD9, host restriction

## Abstract

NTV is a highly attenuated virus that was created by genetically deleting 26 genes related to host range and virulence from TianTan strain. Since NTV is highly attenuated, it has been used widely as an optimizing viral vector. In this study, we explored the biological characteristics *in vitro* and the host restriction mechanism of NTV. Most cell lines do not support sufficient dissemination and replication of NTV, and in non-permissive cell line HeLa, the replication block of NTV occurred at the translation stage of viral late protein expression. Lack of PKR activity was not sufficient to rescue expression of viral late proteins and replication, even though the phosphorylation level of eIF2α increased in NTV-infected HeLa cells. Moreover, the translation inhibition of NTV in HeLa cells was dependent upon a SAMD9 signaling pathway, as demonstrated by silencing SAMD9 expression with siRNA and observing the colocalization of SAMD9 and AVGs. Reinserting *C7L* or *K1L* into NTV rescued the late viral protein expression and replication of NTV in HeLa cells. Among the genes deleted in NTV, C7L or/and K1L gene was mainly responsible for its replication defect. Protein C7 interacted with SAMD9, which antagonized the antiviral response of SAMD9 to ensure viral protein translation and replication of NTV in non-permissive cell lines. Our finding will serve as a baseline for modification of NTV in future application.

## Introduction

Vaccinia virus (VACV), a double-stranded DNA virus of the poxvirus family, was used as the smallpox vaccine during the worldwide smallpox eradication campaign. VACV is currently being investigated as a viral vector for vaccines against various infectious diseases and in cancer therapy because of its efficacious expression of large foreign genes and its ability to induce specific long-term protective humoral and cellular immune responses (Gomez et al., 2011). Despite the advantages, the safety of VACV remains to be ameliorated as pustules and neurotoxicity caused by VACV infection has been reported (Ober et al., 2002). Thus, researchers have developed replication-deficient modified vaccinia virus, which was produced by natural or genetic attenuation of VACV and was restricted in replication of human and a majority of other mammalian cell lines (Pastoret and Vanderplasschen, 2003).

The attenuated replication-deficient VACV strains widely studied as viral vectors are modified vaccinia virus Ankara (MVA) and NYVAC. MVA was attenuated by growing the vaccinia virus Ankara strain in chicken embryo fibroblasts (CEF) for more than 500 generations, 15% of the parental viral genome was lost during the course of attenuation, including genes associated with immunologic escape and host range (Antoine et al., 1998). MVA expressing heterologous antigens was shown to induce considerable specific T-cell immunogenicity in humans against infectious disorders such as AIDS, malaria, and human papillomavirus-associated cancer (Sutter and Staib, 2003). NYVAC was derived from the vaccinia virus Copenhagen (VACV-Cop) by genetically deleting 18 open reading frames (ORFs), which included genes implicated in host range, virulence and pathogenicity (Tartaglia et al., 1994). Present human clinical trials with NYVAC-based vectors have demonstrated quality, safety, and a high level of immunity against different antigens (Raengsakulrach et al., 1999; Hel et al., 2002).

**Figure 1:**
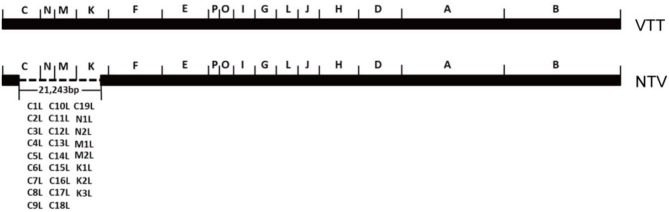
Scheme of deleted genes in NTV genome as compared to VTT. This diagram was created according to reference (Ruan et al., 2006). The deleted genes are indicated.

Another promising attenuated VACA, non-replicating vaccinia virus TianTan (NTV), was derived from vaccinia virus TianTan strain (VTT), which was widely used as the smallpox vaccine in the smallpox eradication campaign in China. NTV was highly attenuated by genetically deleting 26 genes related to host range and virulence (Ruan et al., 2006). Compared with the original virus strain, the genome of NTV lost 21,243 nucleosides-26 genes in total-from the C to K region, including *C1L* to *C19L, N1L* to *N2L, M1L* to *M2L*, and *K1L* to *K3L* (Figure 1). This highly attenuated virus maintains good reproductive capacity in CEFs, while it could no longer replicate or replicated very poorly in most human cell lines, which is the reason why it was called non-replicating vaccinia virus TianTan at that time. NTV showed better safety than VTT as its virulence in mouse and rabbit model was lower (Wang and Ruan, 1991; Guo et al., 2001; Ruan et al., 2006), and recombinant NTV vaccines induced antigen-specific T-cell immune-response against expressed heterologous antigens of HIV, ZIKV, and HPV (Houwen et al., 2006; Qi et al., 2011; Zhan et al., 2019).

Previous studies have reported on the biological properties of MVA and NYVAC, as well as their mechanism of replication inhibition in non-permissive cells. As shown in early studies, the blocked replication of MVA in some mammalian cell lines was a result of blocking virion packaging (Sancho et al., 2002; Gallego-Gomez et al., 2003), whereas in NYVAC, the defective replication was due to the restriction of viral late protein expression (Najera et al., 2006). However, little is known regarding the biological characteristics and replication-defective mechanism of NTV, which might be beneficial for optional vector modification and wider application of this virus vector in the future. In this study, we explored the cellular and biochemical characteristics of NTV and studied its host restriction mechanism. Our findings showed that the replication block of NTV in non-permissive cells occurs at the translation stage of viral late protein synthesis as a result of the intracellular antiviral response of host cells. Among the candidate genes deleted in NTV, we found that loss of *C7L* or *K1L* gene was mainly responsible for the replication defect of NTV, which was associated with the antiviral factor SAMD9. Our finding will serve as a baseline for future modification of NTV as a safer smallpox vaccine with better immunogenicity or a viral vector using for vaccines against other pathogens and in cancer therapy.

## Materials and Methods

### Cells and Viruses

Primary chick embryo fibroblasts (CEFs) were prepared from 8-days-old chicken embryos. MRC-5 and RK13 cells were purchased from China Center for Type Culture Collection (CCTCC). MRC-5 were grown in Minimum Essential Medium Eagles with Earle's Balanced Salts (MEM-EBSS) supplemented with 10% fetal bovine serum (FBS). Other cells were grown in Dulbecco's modified Eagle's medium (DMEM) supplemented with 10%FBS. VTT was provided by National Vaccine and Serum Institute and NTV was from our laboratory.

All viruses were purified by 36% sucrose cushions and tittered by plaque assays in CEFs.

### Construction of NTV-C7L and NTV-K1L

NTV-C7L and NTV-K1L were constructed by reinserting *C7L* or *K1L* gene into VACV TK fragment under the control of the early promoter P7.5. The *C7L* gene was obtained by PCR of genomic VTT DNA using the following set of primers: 5′-CGGGATCCACCATGGGTATACAGCACGAATTC (BamH1 site underlined) and 5′-CGGGATCCCCGGGTTAATCCATGGACTCATAATC (BamH1 site underlined). The *K1L* gene was obtained by PCR of genomic VTT DNA using the following set of primers: 5′-CGGGATCCACCATGGATCTGTCACGAATTAAT (BamH1 site underlined) and 5′-CGGGATCCCCGGGTTAGTTTTTCTTTACACAAT (BamH1 site underlined). The DNA fragments containing *C7L* or *K1L* gene under the control of the P7.5 promoter were amplified from pJET1.2 by PCR and digested with restriction endonucleases BamHI and cloned into pJSC11LacZ vector previously digested with BglII and SmaI. CEFs were infected with NTV at an MOI of 0.01 pfu/cell, and then transfected with either the plasmid pJSC11lacZ-7.5C7L or pJSC11lacZ-7.5K1L using X-tremeGENE HP DNA Transfection Reagent (Roche) according to the manufacturer's instructions. Recombinant NTV viruses containing *C7L* or *K1L* gene were selected by consecutive rounds of plaque purification in CEFs stained with X-gal (5-bromo-4-chloro-3-indolyl-β-D-galactoside). The purity of NTV-C7L and NTV-K1L was confirmed by PCR and DNA sequence analysis.

### Immunostaining of Virus Plaques

The target cells were grown in 6 well plates to 90% confluency and infected with viruses detected at a multiplicity of infection (MOI) of 0.005 pfu/cell. After absorption for 2 h, cells were washed with culture medium three times and supplemented with DMEM with 2%FBS. And then incubated at 37°C. 24 h later, cells were washed with phosphate buffered saline (PBS) for three times and fixed with cold methanol/acetone solution (1:1) for 20 min and then incubated for 1 h at room temperature with PBS containing 3% FBS and then 1 h at 37°C with rabbit anti vaccinia virus antiserum (generated in our laboratory) diluted (1:300) with PBS containing 3% FBS. Cells were then washed by PBS for 5 times and incubated with HRP labeled goat anti rabbit antibody for 1 h at 37°C. After incubation, cells were washed with PBS for 5 times and then visualized using a DAB reagent kit (Solarbio, DA1010).

### Virus Replication *in vitro*

Cells were grown to monolayers in 12-well plates and infected with viruses detected at a multiplicity of infection (MOI) of 0.01 pfu/cell. After absorption for 2 h, cells were washed with culture medium three times and supplemented with DMEM with 2%FBS. Infected cells were harvested at 0, 12, 24, 48, and 72 h p.i. and were tittered by plaque assay in CEFs after freeze-thawing for three times. Data were acquired by three independent replicate experiments. Virus growth kinetic curves were graphed using Graphpad Prism 6.0.

### Western Blot Analysis

HeLa cells were grown to monolayers in 25-cm^2^ culture bottles and mock infected or infected with viruses detected at an MOI of 5 pfu/cell. After absorption for 2 h, cells were washed with culture medium three times and supplemented with DMEM with 2%FBS. Cell lysates were harvested at different hours p.i. and used for western blot analyses. Cells were washed by cold PBS for three times, and mixed with IP (Beyotime, P0013) on ice for 20 min, then the intermixture was collected into an eppendorf tube for centrifugation. supernatant was collected after centrifugation and were mixed with 6X protein loading buffer.

For Western blot analyses, total cell extracts were boiled for 5 min and proteins were fractionated by SDS-PAGE. Following electrophoresis, proteins were transferred to nitrocellulose membranes using a semidry blotting apparatus (bio-rad). The filters were blocked overnight with TBS containing non-fat dry milk at 5% at 4°C, and then incubated with respective primary antibodies at room temperature for 2 h. Primary antibodies were detected with secondary antibodies conjugated to IRDye Infrared Dyes using an Odyssey Infrared Imaging System (Li-Cor Biosciences).

The detection antibodies were as follows: VACV mouse polyclonal E3(1:500), C7(1:50), K1(1:100), G8(1:100), A17(1:200), A27(1:500), and F17(1:100) were generated in our laboratory; rabbit monoclonal eIF2α(1:1000, Cell Signaling Technology, 5324S); rabbit monoclonal phospho-eIF2α (Ser51) (1:500, Cell Signaling Technology, 3398S); rabbit monoclonal βactin (1:1000, Cell Signaling Technology, 4970S); rabbit monoclonal SAMD9 (1:1000, Atlas Antibodies, HPA021319); mouse monoclonal PKR (1:100, R&D, MAB1980); anti-mouse IgG (H+L), HSA, DyLight 800 labeled (1:10000, KPL, 5230-0415); anti-rabbit IgG (H+L), DyLight 680 labeled (1:10000, KPL, 5230-0402).

### Virus Entry

HeLa cells were seeded in 12-well plates and incubated overnight at 37°C. Cells were chilled for 10 min at 4°C and then infected with VTT or NTV at a MOI of 0.01 pfu/cell. Viruses were allowed to adsorb to cells for 1 h at 4°C in DMEM. Unattached viruses were removed by washing and the infection continued at 37°C for another hour. The infected cells were harvested and the viral titer was determined by plaque assay. Data were acquired by three independent replicate experiments.

### DNA Replication

HeLa cells were grown into monolayers in 6-well plates, and were infected with VTT or NTV at an MOI of 0.01 pfu/cell. After absorption for 2 h, cells were washed with culture medium three times, and then DMEM with 2%FBS was added. At 0, 4, 8, and 16 h p.i., infected cells were harvested. Total DNA was extracted from the cell lysates. Real-time PCR was set up using 5μl DNA and TaqMan^R^ Universal PCR Master Mix with Probe-Fam OPE9L-P (sequence: 5′-FAM-CAGGCTACCAGTTCAA-MGBNFQ-3′) and Primer-F OPE9L-F (sequence: 5′ GAACATTTTTGGCAGAGAGAGCC−3′) and Primer-R OPE9L-R (sequence: 5′- CAACTCTTAGCCGAAGCGTATGAG−3′). Amplification conditions were as follows: 95°C for 10 min, followed by 40 cycles at 95°C for 30 s, 60°C for 1 min and a final extension at 72°C for 10 min. Data were acquired by three independent replicate experiments. The relative amount of DNA was calculate by 2^−ΔΔCT^ method, and were normalized to actin.

### DNA Replication Inhibition

To observe the global inhibition of late viral protein expression, cells were incubated in DMEM containing 40 μg/ml 1-β-D-arabinofuranosylcytosine (AraC, Sigma Aldrich) for 30 min. AraC remained present in medium during the adsorption phase of virus infection, in which cells were infected at an MOI of 10 pfu/cell, and for the duration of infection. Cell lysates were harvested and detected by western blot as described above.

### RT-PCR

HeLa cells were grown into monolayers in 6-well plates, and were infected with VTT or NTV at an MOI of 5 pfu/cell. After absorption for 2 h, cells were washed with culture medium three times and supplemented with DMEM with 2%FBS. At 0,4,8,12, and 24 h p.i., infected cells were harvested. Total RNA was extracted from cell lysates and reverse transcribed into cDNA by RT-PCR using 2 g of total RNA and SuperScript III reverse transcriptase (Invitrogen) for cDNA synthesis according to the manufacturer's protocol. Specific primers, cDNA and SYBR green master mix were used in a real-time PCR reaction to detect gene expression. Amplification conditions were as follows: 95°C for 10 min, followed by 40 cycles of 95°C for 15 s, 60°C for 25 s, and 72°C for 25 s, and a final extension at 72°C for 10 min (Ohn et al., 2008). Data were acquired by three independent replicate experiments. The relative amount of mRNA was calculated by 2^−ΔΔCT^ method, and were normalized to actin.

Primers used are as follows:

A17L-F: 5′-CGTAAATACATTGATTGCCAT-3′,
A17L-R: 5′-TCTATTGCCTCTTACTAGCTT-3′,
A27L-F: 5′-TCCAAATTAGTTAGCCGTTGT-3′,
A27L-R: 5′-CGCGAAGCAATTGTTAAAGCC-3′
F17R-F: 5′-TTAGAACTGTAGAATGCGAAG-3′
F17R-R: 5′-TATTCATTCTCTCGCATCTGG-3′
βactin-F: 5′- CAACTCTTAGCCGAAGCGTATGAG -3′
βactin-R:5′-AGGATGGCGTGAGGGAGAGC-3′.

### Quantification of eIF2α Phosphorylation

The signal intensity of phosphorylated eIF2**α** was quantified using Image J 1.8.0 software. The intensities of phosphorylated eIF2**α** were normalized to the intensities of eIF2**α** in respective samples. An increase or decrease in phosphorylation was calculated at 4, 8, and 16 h p.i. relative to mock infected HeLa cells. Each measurement represents the results from three independent experiments.

### Transfection of siRNAs

Oligofectamine (Invitrogen, 12252011) was used to transfect HeLa cells with small interfering RNAs (siRNAs), including siRNAs targeting human SAMD9 and human PKR (synthetized by Sangon) and control respectively, at a final concentration of 20 nM according to the manufacturer's protocol. At 48 h post transfection, the cells were harvested for western blot analysis in order to determine the level of protein knockdown. Cells were also infected with NTV to detect the expression of viral late proteins and viral replication.

Target sequences:

SAMD9-1: 5′-CAAUAUAGCUGGUUAUCAA-3′;
SAMD9-2: 5′-GAACAGGUAACCAGUUUAA-3′;
SAMD9-3: 5′-GGAUGUAAAUCAGUGGUUA-3′;
PKR: 5′-P-ACUUUGUCUAGUUUCUCGCUU-3′.

### Immunofluorescent Staining and Image Capture Using Confocal Microscopy

HeLa cells were seeded onto a cover glass (Thomas Scientific), and then mock treated or infected with VTT or NTV at an MOI of 5 pfu/cell the next day. 16 h p.i., cells were fixed using 4% paraformaldehyde for 10 min at room temperature followed by washing with PBS for three times. Cells were blocked with PBS with 2% goat serum and 0.2% Triton-X-100 for 15 min at room temperature followed by washing with PBS. Primary antibodies, including G3BP1 (1:50, Santa Cruz, sc-365338) and SAMD9 (1:500, Atlas Antibodies, HPA021319) were diluted in PBS with 2% goat serum and incubate on the cover glass at room temperature for 1 h followed by washing with PBS for five times. Secondary antibodies, Fluorescein-Conjugated goat anti-mouse IgG (1:100, ZSGB-BIO, ZF-0312) and rhodamine (TRITC)-conjugated goat anti-rabbit IgG (1:100, ZSGB-BIO, ZF-0316) were diluted in PBS with 2% goat serum to incubate at room temperature for 1 h followed by washing with PBS for five times. Cover glasses were then stained with DAPI for 30 min followed by washing with PBS for five times. A Nikon SP8 confocal microscope (Nikon) was used to observe the fluorescent staining and images were achieved using a 63 × oil objective lens controlled by LAS AF 2.6.0 software.

### Coimmunoprecipitation (co-IP)

HeLa cells were mock treated or infected with NTV or NTV-C7L at an MOI of 5 pfu/cell. 24 h p.i., cell lysates were harvested by IP and rotated with C7 antibody (1:50) at 4°C overnight. Protein A-agarose (Santa Cruz, sc-2001) was washed with washing buffer (50 mM Tris-Cl [pH 7.4], 150 mM sodium chloride, 0.1% NP-40) for three times and rotated with the cell lysate-antibody compound at 4°C for 6–8 h. The agarose was then washed 3 times to remove excess antibody followed by mixing with 6X protein buffer and boiling for 5 min. The agarose samples were then analyzed by western blot.

## Results

### Host Range of NTV Replication and Dissemination

**Figure 2:**
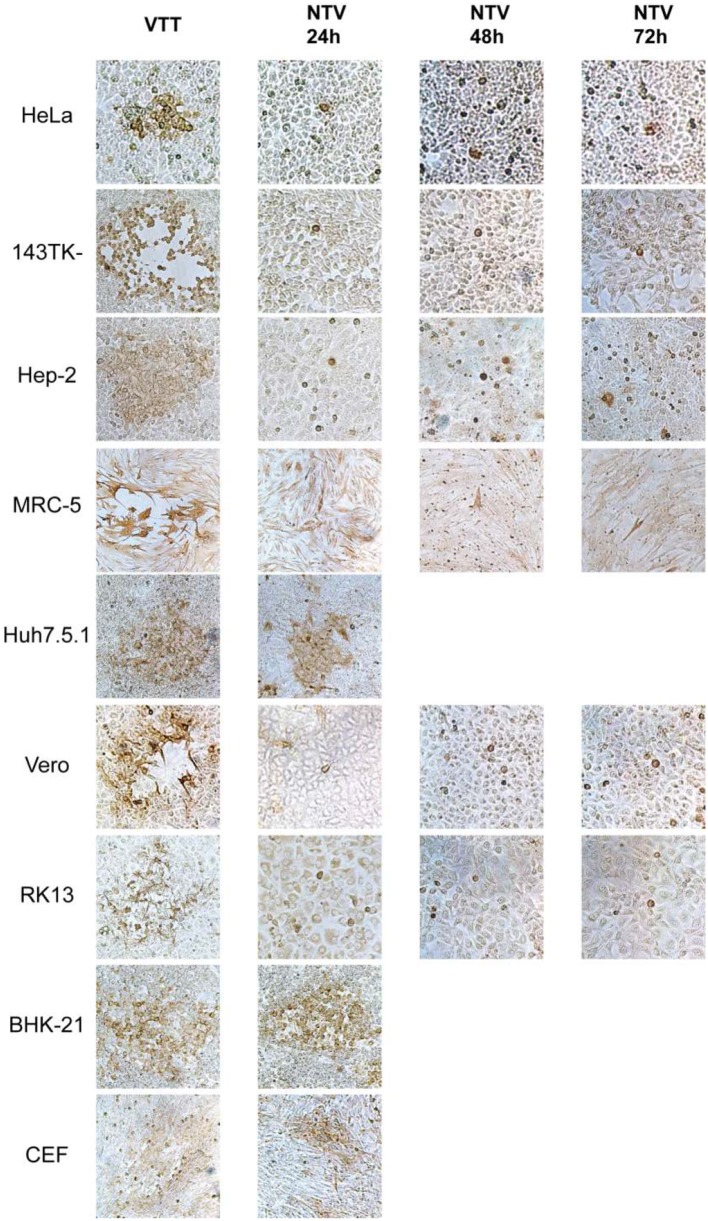
Cell-to-cell dissemination of NTV. The indicated cells were infected with NTV or VTT at an MOI of 0.005, fixed at 24, 48, or 72 h p.i. and immunostained with anti-VACV antibody, and then visualized using DAB reagent kit. VTT was used as control. All images were taken at 200X magnification.

In order to explore the host-range properties of NTV, we used sensitive and specific immunochemical staining to measure virus replication in various cell lines. Primary CEFs and eight other cell lines from various host origins were infected with NTV at an MOI of 0.005. Viral dissemination was assessed using a immunochemical staining method with rabbit polyclonal anti-VACV serum in order to identify cells that expressed VACV protein. We used the parental vaccinia virus TianTan strain (VTT). In the CEF cell line, NTV produced clearly stained foci which contained approximately over 100 cells, and were only slightly smaller than that produced by VTT (Figure 2). Notably, plaques with gaps or holes in its center at 24 h post-infection (p.i.) were observed in NTV-infected Syrian baby hamster kidney BHK-21 cells, but were not observed in other mammalian cells. NTV also produced clear plaques in human hepatitis Huh7.5.1 cells at 24 h p.i. (Figure 2), and the plaque sizes were only a little smaller than those produced in the same cells infected with VTT, indicating that Huh7.5.1 supported dissemination and replication of NTV. Single-stained cell were observed predominantly in NTV-infected human cell lines including HeLa, hep-2, 143TK^−^, and MRC-5, and monkey cell line Vero, and rabbit cell line RK13 (Figure 2) at 24 h p,i, At 72 h p.i., foci containing <5 stained cells were occasionally observed, suggesting that NTV was not able to efficiently spread among those cell lines.

**Figure 3:**
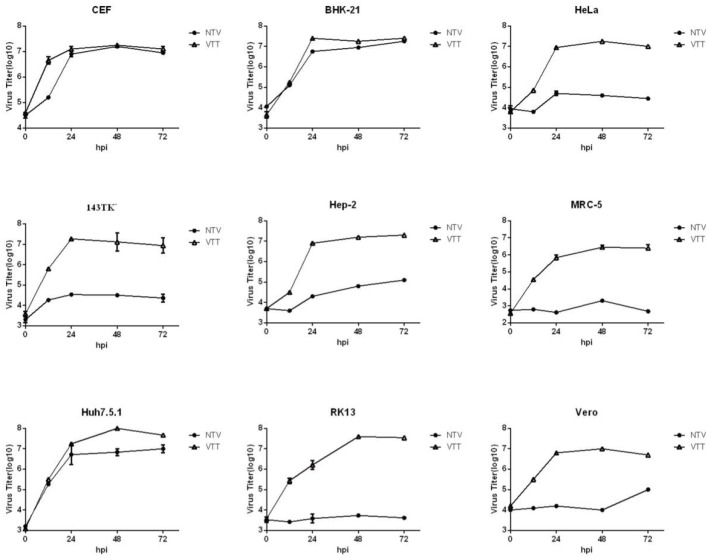
Growth curves of NTV *in vitro*. The indicated cells were infected with NTV or VTT at an MOI of 0.01, and harvested at 0, 12, 24, 48, and 72 h p.i. and tittered by plaque assay in CEFs after freeze-thawing for three times. VTT was used as control. Data were acquired by three independent replicate experiments. Virus growth kinetic curves were graphed using Graphpad Prism 6.0.

To further investigate the ability of viral replication and dissemination in different cell lines, we determined the growth kinetics curves of NTV under multistep growth conditions. Cell lines mentioned above were infected at a low MOI of 0.01 with NTV or VTT. The infected cells were harvested at 0, 12, 24, 48, and 72 h p.i. and were tittered in CEF cells. In most human cell lines, including HeLa, 143TK^−^, Hep-2, and MRC-5 (Figure 3), the titers of NTV remained constant or decreased after the absorption period; thus, the failure of these cells to support replication of NTV was predictable. Additionally, NTV was not able to replicate efficiently in other mammalian cell lines, including Vero and RK13 (Figure 3). Interestingly, Huh7.5.1 was the only infected human cell line that had a significant increase in NTV titers, and the yield was slightly lower compared to that of parental strain VTT. We also found that in BHK-21 (Figure 3), the titer of NTV reached to over 10^7^ pfu/ml at 72 h p.i., which was the highest titer of NTV in all cell lines tested. In primary CEF cells, the replication kinetics of NTV was similar to that of VTT. These results were supported by the viral cell-to-cell dissemination analysis described above. Among all cells above, VTT and NTV had the highest viral titer at 72 h p.i. in Huh7.5.1 and BHK−21, respectively, while the lowest fold increase in viral titers was in MRC-5, as shown in Table 1. Cells were classified into three categories, permissive cells (>25-fold replication), semi-permissive cells (1-25-fold replication), and non-permissive cells (<1-fold replication) based on the yield of virus at 72 h p.i. (Carroll and Moss, 1997). Among the cells tested above, CEF, BHK-21, and Huh7.5.1 were permissive cells, HeLa, 143TK^−^, RK13, and MRC-5 were non-permissive cells and others were semi-permissive cells with a low titer of progeny viruses.

**Table 1:** Replication and spread of NTV and VTT in various cell types.

**Cell lines**	**Species**	**NTV**		**VTT**	
		**Virus spread^**a**^**	**Virus replication^**b**^**	**Virus spread**	**Virus replication**
HeLa	Human	+	0.89 NP	+++	177.83 P
143TK^−^	Human	+	0.82 NP	+++	192.75 P
Hep2	Human	+	4.47 SP	+++	707.95 P
Huh7.5.1	Human	+++	354.81 P	+++	1659.59 P
MRC-5	Human	+	0.02 NP	+++	88.1 P
Vero	African green monkey	+	3.55 SP	+++	177.83 P
RK13	Rabbit	+	0.15 NP	+++	1258.93 P
BHK-21	Syrian hamster	+++	707.95 P	+++	1122.02 P
CEF	Chicken embryo	+++	281.84 P	+++	446.68 P

a *Virus spread as visualized by immunostaining after 72 h. -, no stained cells; +, foci of 1–4 stained cells; ++, foci of 5–25 stained cells; +++, foci of >25 stained cells*.

b *Virus replication (fold increase in virus titer) determined by dividing the virus yield at 72 h by the practical input titer. Cell lines were classified into permissive (P, > 25-fold increase), semi-permissive (SP, 1–25-fold increase) and non-permissive (NP, <1-fold increase) cells*.

A detailed summary relating to NTV dissemination and replication in the 9 cell lines is shown in Table 1.

### NTV Replication Was Blocked at the Stage of Viral Late Gene Translation

The intracellular replication of VACV is regulated in a cascade manner (McFadden, 2005). To explore the step at which NTV replication block occurs in the viral life cycle, we analyzed the intracellular life cycle of NTV in non-permissive cell HeLa by detecting each essential stage of viral replication p.i.

**Figure 4:**
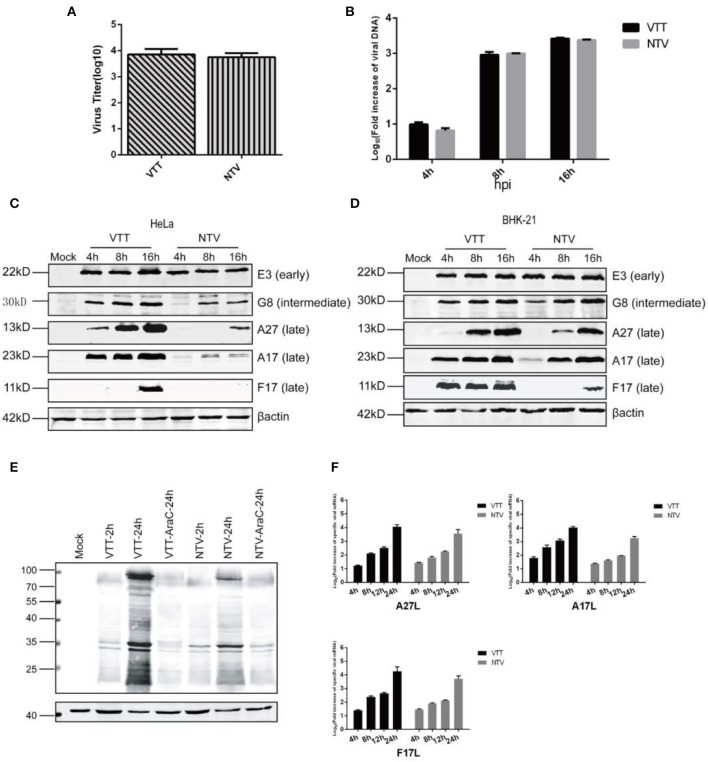
Molecular analyses of the intracellular life cycle of NTV. **(A)** Monolayers of HeLa cells were incubated at 4°C for 10 min, infected with NTV or VTT at an MOI of 0.01, and then incubated at 4°C for 1 h. Cells were washed with DMEM and incubated at 37°C for 1 h, then harvested and tittered by plaque assay in CEFs. **(B)** HeLa cells were infected with VTT or NTV at an MOI of 0.01. The cells were harvested at 0, 4, 8, and 16 h p.i., viral DNA was isolated and measured by real-time PCR using TaqMan^R^ Universal PCR Master Mix. Primer sets of VACV OPE9L were designed to selectively amplify VACV DNA. Real-time PCR for OPE9L at each time point for each infection was conducted in triplicate. The Livak (2^−ΔΔCT^) method was used to calculate the relative fold increase in viral DNA during VTT or NTV infection. The DNA level at 2 h p.i. of each sample was as control, which was set artificially as 1-fold. The curve shown is a summary of results from three independent experiments, and error bars represent ± SD. **(C)** Monolayers of HeLa cells were mock infected or infected at an MOI of 5 with VTT or NTV. Cell extracts were harvested at 4, 8, and 16 h p.i. and analyzed by western blot using antibodies against specific viral early protein E3, viral intermediate protein G8 and viral late proteins A27, A17, and F17. **(D)** Monolayers of BHK-21 cells were mock infected or infected at an MOI of 5 with VTT or NTV. Cell extracts were harvested at 4,8,16 h p.i. and analyzed by western blot using antibodies against viral early protein E3, viral intermediate protein G8 and viral late proteins A27, A17, and F17. **(E)** Monolayers of HeLa cells were mock treated or pretreated with AraC at 40 μg/ml, then mock infected or infected at an MOI of five with VTT or NTV. Cell extracts were harvested and analyzed by western blot using rabbit anti-VACV serum. **(F)** HeLa cells were infected with VTT or NTV at an MOI of 5, at 0, 4, 8, 12, and 24 h p.i. Total RNA was extracted and reverse transcribed to produce cDNA. The transcript levels of *A27L, A17L*, and *F17R* were assessed by real-time PCR using SYBR green mix system. The Livak (2^−ΔΔCT^) method was used to calculate the relative fold increase in viral mRNA during VTT or NTV infection. The cDNA level at 2 h p.i. of each sample was as control, which was set artificially as 1-fold. Curve shown is a summary of results from three independent experiments, and error bars represent ± SD.

First, in order to examine whether entrance influences viral replication, we compared the entry of NTV and VTT. HeLa cells were infected with NTV or VTT, incubated at 4° for 1 h, and then unattached virus was removed by washing with DMEM. The cells were transferred to 37° for another hour and then harvested in order to assess the titer of virus that enters into the cells. As shown in Figure 4A, the virus titer in host cells was similar between NTV and TTV. This result indicated that the replication block of NTV did not occur at the stage of viral particles entering the host cells.

The early genes of VACV are transcribed and translated immediately after the virus enters host cells and releases viral DNA (Broyles, 2003). Thus, we speculated if the expression of early genes was blocked. As reported previously (Meng et al., 2008), *C7L* is required to maintain *E3L* expression in HeLa cells. *C7L* is deleted in NTV, and we monitored E3 protein level, the product of VACV early viral gene *E3L* by western blot in NTV-infected HeLa cells. This result showed that the E3 protein was detected, and the level of *E3L* gene expression was similar to VTT (Figure 4C), which excludes early gene expression as the reason of the defective replication of NTV.

After early gene expression, VACA virus begins replicating its DNA, which is a prerequisite for VACV intermediate gene and consequent late gene expression (Keck et al., 1990). To analyze if viral DNA replication of NTV is inhibited, we determined the viral DNA synthesis by isolating total DNA from HeLa cells infected with NTV or VTT at 0, 4, and 8 h p.i. the DNA amounts were detected by real-time PCR, which showed that while the DNA levels of NTV were slightly lower than VTT at 4 h p.i., NTV DNA replication increased with over time and was similar to that of VTT at 8 and 16 h p.i. in HeLa cells (Figure 4B). This suggested that the replication of viral DNA was favorable and was not responsible for the host restriction of NTV.

Intermediate and late genes are expressed After VACV DNA replication. The production of viral intermediate and late genes play an important role in the maturation, packaging, and transportation of viral particles and intermediate proteins are essential for late genes transcription; thus, we detected VACV intermediate protein G8, and late proteins A27, A17, and F17 by western blot in cell lysates of non-permissive HeLa cells or permissive BHK-21 cells infected with NTV or VTT. Intermediate viral protein G8 was efficiently detected in cells infected with both NTV and VTT, but the expression level of G8 in NTV-infected HeLa cells was decreased as compared to that of VTT (Figures 4C,D). All three late viral proteins monitored were detected in VTT-infected HeLa cells, which was consistent with its efficient replication. In NTV infected HeLa cells, only A27 were detected at 16 h p.i., but the stripe was clearly narrowed compared to that of VTT. However, late protein A17 and F17 were barely detected in NTV-infected HeLa cells (Figure 4C), which suggested that the block of viral late protein expression was the reason for the abortive replication. In contrast, the expression level of these three late proteins was similar to that of VTT at 16 h p.i. in NTV-infected permissive BHK-21 cells (Figure 4D).

In order to observe the overall expression of intermediate and late proteins, we next inhibited DNA replication in NTV-or VTT-infected HeLa cells by using the DNA replication inhibitor 1-β-D-arabinofuranosylcytosine (AraC) and detected viral protein expression by western blot. Early gene expression before DNA replication in NTV was similar to that of VTT, while a global inhibition of protein synthesis was observed after viral DNA replication in NTV-infected HeLa cells, as shown in Figure 4E. This suggested that, except for intermediate protein G8 and the three late proteins detected above, viral intermediate and late protein expression in NTV-infected HeLa cells was reduced or inhibited broadly.

Finally, we monitored the transcription level of the three late viral genes by RT-PCR. RNA of NTV-or VTT-infected HeLa cells was isolated at 4, 8, 12, 24 h p.i. and reverse transcribed by RT-PCR. As shown in Figure 4F, we found that although the transcription level of late genes *A27L, A17L*, and *F17R* of NTV was a little bit lower than VTT at each time point, transcription level of the three late genes in both viruses continuously increased over time, indicating highly reiterative transcription of viral DNA templates in NTV-infected HeLa cells. These results suggested that the reduction of intermediate protein G8 was not enough to block the transcription of late viral genes, and the block of late viral gene expression did not occur at the transcription stage but possibly at the translation stage.

Altogether, our data suggest that inhibition of NTV late protein expression associated with viral late gene translation is responsible for the host restriction of NTV.

### Lack of PKR Activity Was Not Sufficient to Rescue Expression of NTV Late Genes in Non-permissive Cells

**Figure 5:**
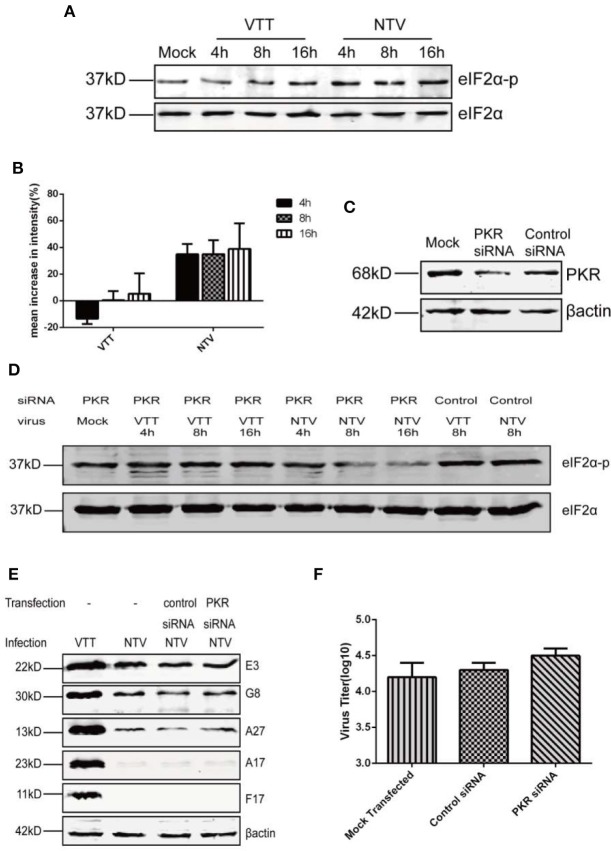
Replication inhibition of NTV was independent of PKR antiviral pathway. **(A)** HeLa cells were mock infected or infected with VTT or NTV at an MOI of five. Cell extracts were harvested at 4, 8, and 16 h p.i. and analyzed by western blot using eIF2α and p-eIF2α antibodies. **(B)** Mean increases in signal intensity of phosphorylated eIF2α at 4, 8, and 16 h p.i. relative to mock infected HeLa cells. **(C)** HeLa cells were transfected with PKR siRNA or control siRNA using oligofectamine, 48 h later, the cell lysates were harvested to detect PKR level by western blot, or **(D)** infected with NTV or VTT at an MOI of five, in order to assess the phosphorylation level of eIF2α or **(E)** infected with NTV at an MOI of 5, in order to assess viral protein at 16 h p.i. or **(F)** infected with NTV at an MOI of 0.01 to detect viral titer at 48 h p.i.

It is well-known that, depending on the stress response, protein kinase influences protein translation via phosphorylation of the α-unit of eIF2, which downregulates the initiation of global translation (Liem and Liu, 2016; McCormick and Khaperskyy, 2017). To further explore what interferes with the translation of viral late genes in non-permissive cells infected with NTV, we monitored the phosphorylation level of eIF2α in NTV-infected HeLa cells by western blot using a specific antibody against phosphorylated eIF2α (Figures 5A,B). We found that the level of eIF2α phosphorylation induced by NTV infection increased at 4, 8, and 16 h p.i., while VTT induced only a slight increase at 16 h p.i.

Phosphorylation of eIF2α by PKR, PERK, and GCN2 has been reported to be associated with viral infection, but only PKR-mediated eIF2α phosphorylation was related to VACV infection (Jordan et al., 2002; Berlanga et al., 2006; Garcia et al., 2007; Backes et al., 2010; Sivan et al., 2018). In order to further confirm whether the increase in eIF2α phosphorylation caused by NTV infection blocked the translation of NTV late viral genes, we reduced PKR expression in HeLa cells using siRNA against PKR (Figure 5C). Comparing to control siRNA separately, eIF2α phosphorylation level was apparently reduced in NTV infected HeLa cells, and slightly reduced in VTT infected cells (Figure 5D). Following we analyzed NTV late protein expression and viral replication (Figures 5E,F). Our results showed that the block of viral protein synthesis was not rescued, and the replication of NTV not recovered in HeLa cells with knocked down PKR expression.

Altogether, our data showed that PKR-mediated phosphorylation of eIF2α was not primarily responsible for the defective late gene expression and replication block of NTV. Furthermore, these results suggested that the restriction of viral late gene translation might be regulated by a PKR-independent antiviral pathway.

### Translation Inhibition of NTV Occurred in a SAMD9-Dependent Antiviral Response Pathway

**Figure 6:**
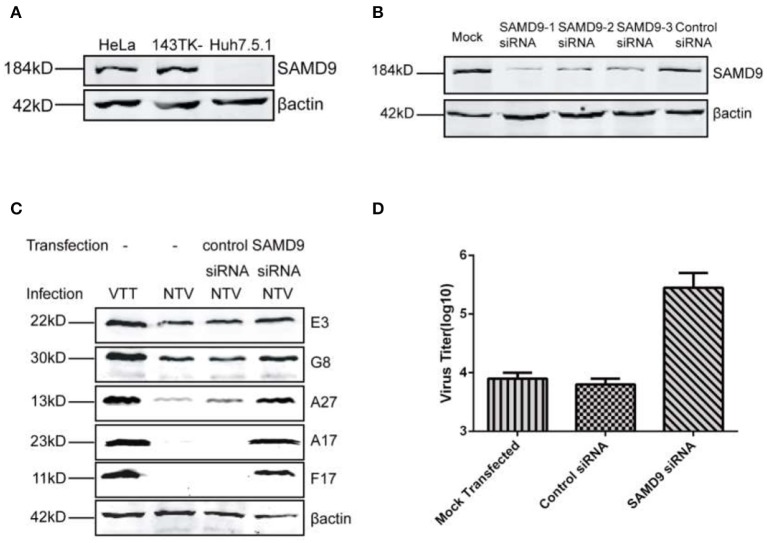
Replication of NTV was inhibited in a SAMD9 dependent antiviral pathway. **(A)** HeLa, 143TK^−^, and Huh7.5.1 cells were harvested, and SAMD9 expression was detected by western blot. **(B)** HeLa cells were transfected with SAMD9 siRNA or control siRNA using oligofectamine, 48 h p.i., cell lysates were harvested to detect PKR level by western blot, or **(C)** infected with NTV at an MOI of five in order to detect viral protein at 16 h p.i. or **(D)** infected with NTV at an MOI of 0.01 to detect viral titer at 48 h p.i.

Previous studies have identified sterile α motif domain-containing protein 9 (SAMD9) as a major restriction factor that blocks replication of VACV mutants lacking both *C7L* and *K1L*. The human host-restriction of the VACV mutants was mediated by an atypical mode of translation inhibition through SAMD9 (Sivan et al., 2015, 2018). In this study we also blocked the expression of SAMD9 in HeLa cells using siRNA against SAMD9 (Figure 6B) and observed if it was able to rescue the translation of viral late genes. We chose one of the three siRNAs to transfect into HeLa cells and detected NTV viral replication and late viral protein expression. We found that the expression of the three late viral proteins monitored was recovered in SAMD9-silenced HeLa cells infected with NTV (Figure 6C) and the viral titer of NTV nearly increased 100-fold at 48 h p.i. (Figure 6D). This data was supported by our results that NTV was capable of replicating in human Huh7.5.1 cells (Figures 2, 3), a cell line lacking SAMD9 expression (Figure 6A).

**Figure 7:**
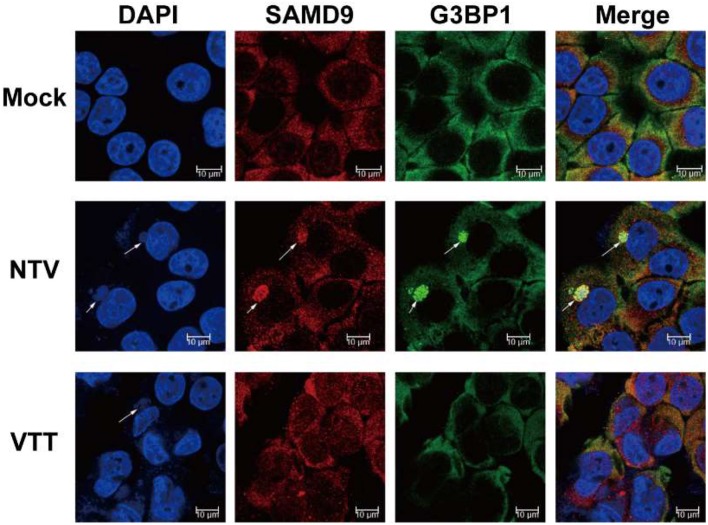
Colocalization of SAMD9 with G3BP1. HeLa cells were mock infected or infected with VTT or NTV at an MOI of 5, 16 h p.i., cells were incubated with specific primary antibodies against SAMD9 and G3BP1 after immobilizing and then incubated with the appropriate secondary antibodies. After incubation with antibodies, the cells were stained with DAPI. Fluorescent images were captured using a Nikon SP8 confocal microscope. Arrowheads are pointing at the viral factor.

Mammalian cell stress responses are very closely intertwined with translation regulation, Antiviral granules (AVGs) formation is a manifestation of translation inhibition and reinforces the antiviral effect of protein synthesis arrest (Liem and Liu, 2016; McCormick and Khaperskyy, 2017). Therefore, to further confirm the interference of SAMD9 with the viral protein translation of NTV, we colocalized SAMD9 with G3BP1 (RAS GTPase-activating protein SH3 domain-binding protein 1), a marker of AVGs forming around the viral factory, We observed that SAMD9 as well as G3BP1 accumulated and formed a granule structure around the viral factories in the cytoplasm of HeLa cells infected with NTV. SAMD9 and G3BP1 were distributed throughout the cytoplasm (Figure 7). Pictures of wider fields of cells are shown in Supplementary Figure 1. We therefore inferred that SAMD9 influenced the viral translation of NTV late genes under non-permissive conditions and participated in the formation of AVGs.

HeLa cells were mock infected or infected with VTT or NTV at an MOI of 5, 16 h p.i., cells were incubated with specific primary antibodies against SAMD9 and G3BP1 after immobilizing and then incubated with the appropriate secondary antibodies. After incubation with antibodies, the cells were stained with DAPI. Fluorescent images were captured using a Nikon SP8 confocal microscope. Arrowheads are pointing at the viral factory.

### Reinserting *C7L* or *K1L* Genes Rescued the Replication Defect of NTV in HeLa Cells and *C7L* Bound With SAMD9, Antagonizing the Function of SAMD9

**Figure 8:**
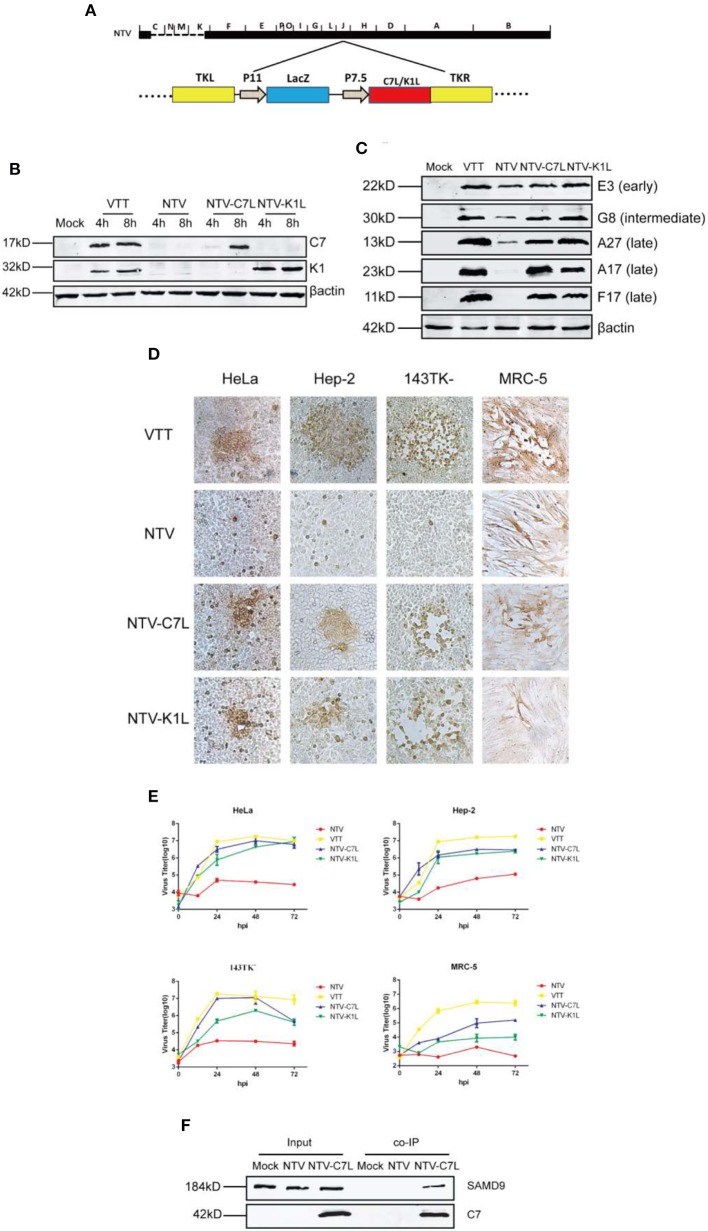
Reinserting vaccinia host range genes *C7L* or *K1L* rescued NTV replication in human cell lines. **(A)** Construction of NTV-C7L and NTV-K1L. NTV-C7L and NTV-K1L were constructed by reinserting *C7L* or *K1L* gene into VACV TK fragment under the control of the early promoter P7.5. **(B)** Identification of NTV-C7L and NTV-K1L construction. Monolayers of HeLa cells were mock infected or infected at an MOI of five with VTT, NTV, NTV-C7L or NTV-K1L. Cell extracts were harvested at 4 and 8 h p.i. and analyzed by western blot using antibodies recognizing viral early proteins E3, C7 and K1. **(C)** Protein expression of NTV-C7L and NTV-K1L. HeLa cells were infected with VTT, NTV,NTV-C7L or NTV-K1L at an MOI of five and harvested at 16 h p.i. Viral proteins were detected as described in Figure 4C. **(D)** Cell-to-cell dissemination of NTV-C7L and NTV-K1L was detected as described in Figure 3. **(E)** Viral replication kinetics were detected as described in Figure 2. **(F)** Co-IP of SAMD9 and protein C7. HeLa cells were mock treated or infected with NTV or NTV-C7L at an MOI of five. Cells were harvested at 24 h p.i., incubated with C7 antiserum overnight and then incubated with protein A for 4 h. Then protein A was washed and SAMD9 and C7 were detected by western blot.

NTV was generated by deleting 26 genes, including host range genes *C7L* and *K1L*, which were mainly responsible for the replication defect of NTV in most human cell lines. SAMD9 is known to be a critical poxvirus restriction factor in human cell lines, and K1 or C7 protein was shown to bind with human SAMD9 and antagonize its antiviral activity (Li et al., 2010; Meng et al., 2018). Therefore, we wondered if reinsertion of *C7L* or *K1L* into NTV genome could rescue some of the biological properties of NTV. To this aim, we supposed to construct two NTV recombinants, NTV-C7L and NTV-K1L. We had constructed a modified NTV strain (NTV11LacZ7.5), which contains beta-galactosidase marker gene inserted at TK locus of NTV. It could not grow in human HeLa cells as same as NTV (Supplementary Figure 2), which indicates that the disruption of J2R doesn't restore the growth of NTV in HeLa cells. Therefore, we constructed NTV-C7L and NTV-K1L by reinserting *C7L* or *K1L* gene back into NTV genome at TK locus of NTV (Figure 8A), and measured the expression of viral proteins and the replication of the two recombinant virus strains in infected HeLa cells (Figure 8B). All viral proteins detected were expressed as efficiently as VTT (Figure 8C), indicating that the block of translation was rescued by reinserting *C7L* or *K1L*. Next, we detected the cell-to-cell dissemination and replication kinetics of NTV-C7L and NTV-K1L in NTV non-permissive and semi-permissive human cell lines. As Figures 8D,E show, in HeLa, Hep-2, and 143TK^−^ cells, NTV-C7L and NTV-K1L restored replication ability and produced clear stained foci smaller in size than those formed by VTT, NTV-K1L had lower growth rate compare with NTV-C7L in infected 143TK^−^ cells. Interestingly, in human diploid cell line MRC-5, NTV-C7L produced plaque that was smaller in size than those formed by VTT, and recover the replication capability, the viral titer was increased ~100-fold. While, NTV-K1L formed foci <5 stained cells, and nearly no progeny viruses were produced in infected MRC-5 cells (Figures 8D,E).

As mentioned above, SAMD9 played an important role in the intracellular antiviral response against NTV. However, the mechanism through which C7 or K1 protein antagonizes the function of SAMD9 and rescues viral protein expression and NTV replication is not fully understood. It was suggested by co-immunoprecipitation (co-IP) studies that SAMD9 independently interacted with both C7 or K1 proteins in HeLa cells transfected with the plasmid expressing C7 or K1 protein (Sivan et al., 2015). Here, we detected binding of SAMD9 with C7 or K1 protein by co-IP in HeLa cells infected with NTV-K1L or NTV-C7L virus. Lysates of mock, NTV, NTV-C7L or NTV-K1L infected HeLa cells were harvested at 24 h p.i. and incubated with polyclonal antiserum against C7 or K1. Protein A was incubated with the lysate-antiserum conjugation, and then the protein A beads were isolated and washed. SAMD9 was detected by western blot analysis of the beads. As shown in Figure 8F, SAMD9 was monitored in the protein A beads of NTV-C7L-infected HeLa cell lysate, suggesting that the protein-protein interaction with human SAMD9 and C7 was existent. However, we did not monitor the co-IP of K1 protein with SAMD9 in NTV-K1L-infected HeLa cells (data not shown), the polyclonal antiserum against K1 used in this study might not be suitable for this assay.

## Discussion

VACV initially played an essential role in the campaign against smallpox in the middle of the twentieth century, and then it is studied as a viral vector. However, safety problems appeared during the application of VACV vector, which inspired the creation of replication-deficient vaccinia virus through the way of natural or genetic attenuation. NTV strain was developed by genetically deleting 26 genes related to host range and virulence from VTT (Ruan et al., 2006). NTV has been shown to exhibit good safety and immunogenicity, as demonstrated by targeting diverse exogenous antigens in vaccinated animals (Houwen et al., 2006; Qi et al., 2011; Zhan et al., 2019). In order to further improve the performance of NTV as a gene transfer or vaccine vector by rationally modifying the NTV viral genome, we explored the cellular and biochemical properties and replication-defective mechanism of NTV.

According to the replicate cycle of VACV (Broyles, 2003; McFadden, 2005; Moss, 2006), we carefully detected the entry, early gene expression, DNA replication and expression of intermediate and late genes of NTV life cycle in order to analyze the replication-defective mechanism. We found that the expression of late viral genes of NTV was severely inhibited in non-permissive HeLa cells as compared with parental virus strain VTT. Among the three late viral protein detected, A17 is an essential component of the immature virus (IV) membrane and plays an important role in the formation of IMV membranes (Unger et al., 2013), A27 plays an important role in the transportation of intracellular mature virion (IMV) (Smith et al., 2002), F17 is a major component of the quasi-brick-shaped core structure, and inhibition of its expression would destroy the morphology of viral particles (Wickramasekera and Traktman, 2010). Therefore, we speculated that the inhibition of late gene expression was the main cause of the abortive replication of NTV. This was confirmed by the morphogenesis of NTV in non-permissive HeLa cells. Fewer IVs were detected in the cytoplasm compared with VTT (data not shown), which suggested that the NTV morphogenetic program was blocked at the stage of immature virus formation. Thus, we speculated that mature virions did not form as scheduled, which in turn led to the abortion of NTV replication, as evidenced by the inhibition of late viral protein synthesis. This phenomenon was similar to NYVAC, as these two attenuated VACV strains both lost *C7L* and *K1L* host range genes. The other attenuated VACV strain MVA, which possessed *C7L* gene, expressed late viral genes sufficiently (Najera et al., 2006), The replication block of MVA was due to the inhibition of viral particles packaging (Sancho et al., 2002; Gallego-Gomez et al., 2003). The NTV and NYVAC replication restriction is beneficial to the safety of viral vectors. However, the arrest of late viral protein synthesis is not advantageous for the usage of these viral vectors in a second-generation vaccine against smallpox, since late proteins can induce protective neutralizing antibodies. We also confirmed that reinserting of *C7L* or *K1L* into NTV rescued the expression of late viral genes; thus, the existence of either *C7L* or *K1L* was indispensable for VACV late viral expression in most human cell lines.

Although the transcription of late genes proceeded effectively in NTV infected HeLa cells, the transcription level was slightly lower than that of VTT, this might be the reason of decrease of intermediate protein expression in NTV infected HeLa cells, since intermediate proteins are prerequisite in late protein transcription of VACA. However, decreased intermediate protein expression was sufficient to support late protein transcription in NTV. Since the transcriptional stage of NTV late genes was favorable, we speculated that the arrest of late viral protein expression in NTV occurred at the translation stage of viral gene expression. In order to defend against VACV infection, host cells initiate antiviral responses, which interferes with the global translation of viral proteins and antagonize the replication of viruses (Liem and Liu, 2016). Gilad Sivan et al. hypothesized that it takes time to establish the inhibitory state of translation (Sivan et al., 2018), Our data support this hypothesis, which could explain why the intermediate and late viral gene expression of NTV in non-permissive HeLa cells was inhibited to different extents, and the early viral gene E3 was less affected (Figure 4). However, in Sivan's research, the expression of intermediate viral protein D13 in VACV ΔC7K1-infected HeLa cells was severely inhibited at 8 h p.i., which was not entirely consistent with our results, which might be because of the different virus strains we used.

Currently, two cellular antiviral response programs have been reported to be associated with VACV mutant infection and induced inhibition of translation initiation. One is the PKR-dependent antiviral pathway, and the other is related to intracellular host factor SAMD9. During the PKR-dependent antiviral pathway, the activation of PKR phosphorylates the α-unit of eIF2α to downregulate global translation initiation in the presence of double-stranded RNA (dsRNA) during viral infection (Liem and Liu, 2016). In our results, we observed an increase in the levels of phosphorylated eif2α in NTV-infected non-permissive HeLa cells (Figures 5A,B). However, silencing PKR expression did not recover the viral late genes expression and replication of NTV. This result was supported by the early finding that eIF2α phosphorylation by PKR was not responsible for the restriction of late viral gene expression in murine cells infected with MVA-ΔC7L (Backes et al., 2010). During VACV infection, its early protein E3 is the primary antagonist against PKR-dependent antiviral pathway. Early researches show that E3 antagonize PKR activation by sequestering the activator dsRNA and direct protein-protein interaction with the substrate binding region of PKR (Zhang et al., 2008). As E3L gene remains both in VTT and NTV genome, we supposed that E3 protein was limiting the phosphorylation level of eIF2α which resulted in limited differences between VTT and NTV infection, explaining why we observed there were only small differences between the eIF2α phosphorylation levels of NTV and VTT infection in either primary or PKR silenced HeLa cells.

Sterile Alpha Motif Domain 9 (SAMD9) is known as an anti-neoplastic factor, and its antiviral function was recently demonstrated. A recent study identified SAMD9 as an antiviral factor against VACV by human genome-wide RNA interference screening (Sivan et al., 2015). We monitored SAMD9 expression level in several different human cell lines and found that, SAMD9 expression was deficient in Huh7.5.1 (Figure 6A), the only human cell line in which NTV was able to replicate efficiently, and the fold increase in viral titer reached over 300 (Table 1), indicating that the inhibition of NTV replication was related to SAMD9. We silenced the expression of SAMD9 in HeLa cells, and found that the viral expression and replication of NTV were recovered. Although both host-range genes *C7L* and *K1L* were involved in the inhibition of viral late protein expression induced by SAMD9, the mechanism remains unclear. Recently, a study reported that viral mRNA was sequestered by SAMD9 in C7/K1 deletion mutant-infected HeLa cells. The authors speculated that SAMD9 directly interacted with viral mRNA, instead of translation factors. However, this needs to be further investigated in order to elucidate the exact mechanism (Sivan et al., 2018).

As a result of translation arrest, untranslated mRNPs (messenger ribo-nucleoproteins) bind with G3BP1 and/or TIA1 (T cell-restricted intracellular antigen 1) or TIAR (TIA1-related protein) to form small core aggregates, which can grow and fuse to form larger granules, called stress granules. During poxvirus infection, these stress granules are also called antiviral granules (AVGs) (Simpson-Holley et al., 2011; Wheeler et al., 2016). SAMD9 was demonstrated participate in the formation of AVGs (Liem and Liu, 2016; Sivan et al., 2018). Moreover, we colocalized SAMD9 and G3BP1, a representative component of AVGs, and found that in NTV-infected HeLa cells the two factors accumulated together around the viral factory. The same phenomenon was also observed in VACVΔC7K1 infected HeLa cells (Liu and McFadden, 2015), confirming that the AVGs in C7L and K1L both deleted VACV mutant strain infected cells were formed by the translation initiation block induced by the intracellular antiviral factor SAMD9. In addition, recent reports have suggested that AVGs can function as novel signaling platforms and regulate antiviral response pathways (Onomoto et al., 2012). However, the exact role of AVGs aggregation in the antiviral responses of host cells infected with VACV has not been directly examined and, as such, remains unknown.

Since NTV lost *C7L* and *K1L*, two of the most important VACV host range genes, we reinserted these genes into NTV and constructed NTV-C7L and NTV-K1L, We found that when VACV host range gene *C7L* or *K1L* were reinserted back into NTV, viral late protein synthesis and replication in the most of human non-permissive cell lines were rescued, except for MRC-5, in which NTV-C7L slightly recovered viral replication capacity and the expression level of viral late proteins A17 and A27 of NTV-C7L was detected at levels similar to that of VTT, while the expression level of late protein F17 was much lower with a smeared band observed in the western blot analysis as compared to that of VTT (data not shown). This might be the reason that NTV-C7L replication was lower in infected MRC-5 cell line. However, NTV-K1L remained replication-defective, and the three late proteins were not detected in infected MRC-5 cell line (data not shown). This phenomenon cannot be supported by the fact that in human cell lines protein K1 can bind with SAMD9 to antagonize its antiviral function. This suggested that different intracellular environments might be responsible for this phenomenon. MRC-5, human embryonic lung diploid cells, which is not immortalized and is similar to primary cells, This specific intracellular environment might restrict the replication capacity of viruses, as the replication level of VTT, the parental strain of NTV, was the lowest in MRC-5 as compared to that in human tumor cell lines (Table 1). Since their intracellular environment is more similar to normal cells of the human body, human diploid cell lines are a better system than human tumor cell lines to study biological characteristics of vaccinia viral vectors as potential vaccines against infectious diseases or potential cancer therapies for humans. Hence, it is very important and interesting to explore how MRC-5 cells block late protein expression and replication of NTV-K1L after infection. In the future, we also will explore the toxicity and immunogenicity of NTV-modified strain NTV-C7L and NTV-K1L *in vivo* to find a balance between toxicity and immunogenicity in order to optimize NTV as a better vaccine vector.

A previous study found that C7 or K1 protein interacted with SAMD9 by co-IP in HeLa cells transfected with the plasmid expressing K1 or C7 protein (Sivan et al., 2015). Here we confirmed C7 protein binding with SAMD9 by co-IP in HeLa cells infected with NTV-C7L vaccinia strain, which expressed C7 protein by reinserting *C7L* gene into NTV (Figures 8B,F). Due to this protein-protein binding, which antagonized the function of antiviral host factor SAMD9, viral late protein expression and replication of NTV were recovered in non-permissive human cells (Figures 8C,E), This correlated with the result that SAMD9 expression being inhibited by siRNA rescued the viral late protein expression and abortive infection in HeLa cells infected with NTV (Figure 6). Our results were supported by a previous report which found that poxvirus host-range proteins that share homology with vaccinia virus C7 protein could overcome SAMD9 by forming a unique ‘three-fingered molecular claw’ (Meng et al., 2015). Protein K1 was also confirmed to bind with SAMD9 to antagonize its antiviral function by co-immunoprecipitating SAMD9 with FLAG epitope-tagged K1 protein using a monoclonal antibody against FLAG (Sivan et al., 2015). However, in our co-IP analysis, we did not observe the interaction of SAMD9 and K1 in the HeLa cells infected with NTV-K1L, even K1 protein was not detected in the protein A beads, which captured proteins in the cell lysates incubated with 1:50 dilution of polyclonal antiserum against K1 (data not shown). The reason might be the combination of polyclonal antibody against K1 and K1 protein expressed in NTV-K1L-infected HeLa cells was affected by SAMD9, which might have bound to K1 protein, effectively hiding the epitope of K1. Another possibility might be that our polyclonal antiserum against K1, which was developed by immunization of mice with K1 protein expressed in *Escherichia coli*, does not recognize K1 protein expressed in eukaryotic HeLa cells, since the structure of K1 differs in the two expression systems.

In conclusion, NTV was replication-defective in most human cell lines, and the inhibition of its replication occurred during the translation stage via a SAMD9-dependent antiviral pathway. Reinserting the host range gene *C7L* or *K1L* rescued the replication of NTV by antagonizing the antiviral response of SAMD9. Our data pave a path for the improvement and application of NTV as a gene transfer or vaccine vector by rationally modifying the NTV viral genome.

## Data Availability Statement

All datasets generated for this study are included in the article/Supplementary Material.

## Author Contributions

HT and WT contributed conception and design of the study. YZ, LZ, PH, JR, and PZ contributed investigation and acquisition of the database. YZ wrote the first draft of the manuscript. HT and WT wrote sections of the manuscript. All authors contributed to manuscript revision, read, and approved the submitted version.

### Conflict of Interest

The authors declare that the research was conducted in the absence of any commercial or financial relationships that could be construed as a potential conflict of interest.
