# Supplementary material for: Non-replicating Vaccinia Virus TianTan Strain (NTV) Translation Arrest of Viral Late Protein Synthesis Associated With Anti-viral Host Factor SAMD9
Source: Front Cell Infect Microbiol. 2020 Mar 20;10:116. doi: 10.3389/fcimb.2020.00116 (PMC7098914; doi:10.3389/fcimb.2020.00116)
Supplement: Supplementary file 1 [file Data_Sheet_1.PDF]

Supplementary Material

Supplementary Figures

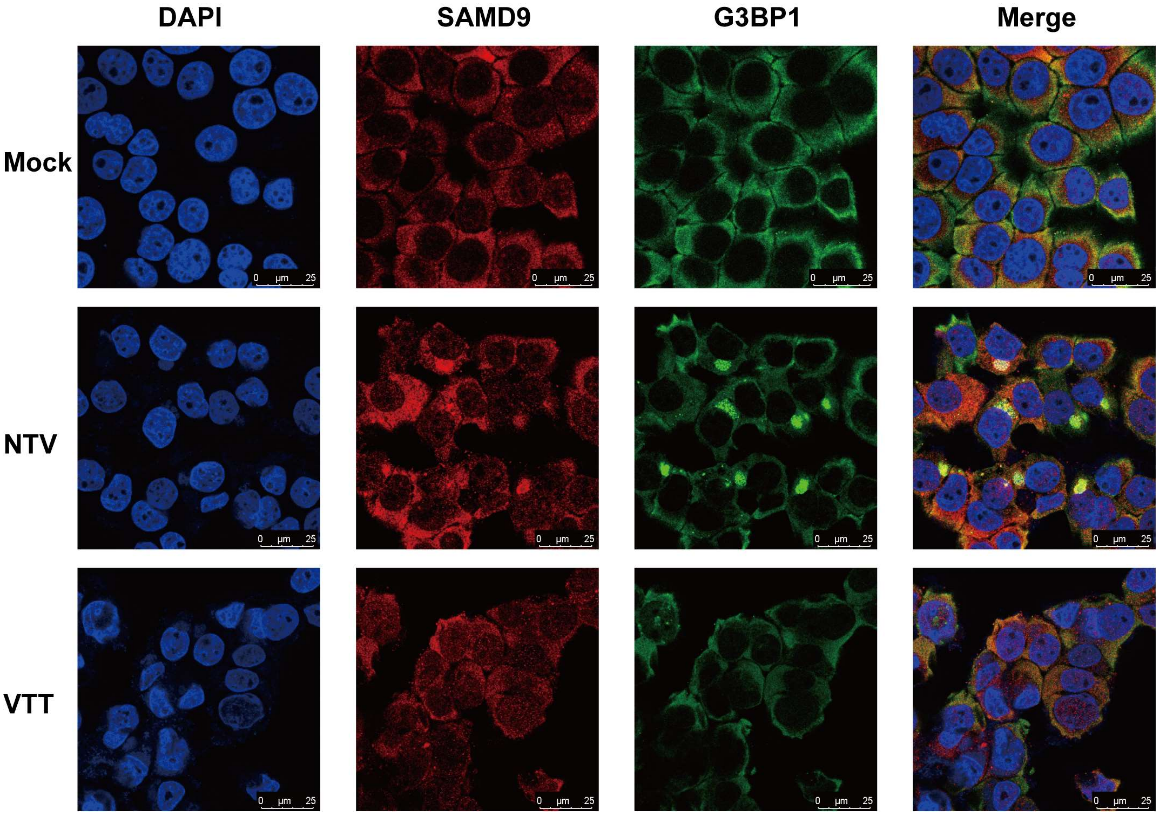

Supplementary Figure 1. Colocalization of SAMD9 with G3BP1.
HeLa cells were mock infected or infected with VTT or NTV at an MOI of 5, 16 hours p.i., cells were incubated with specific primary antibodies against SAMD9 and G3BP1 after immobilizing and then incubated with the appropriate secondary antibodies. After incubation with antibodies, the cells were stained with DAPI. Fluorescent images were captured using a Nikon SP8 confocal microscope.

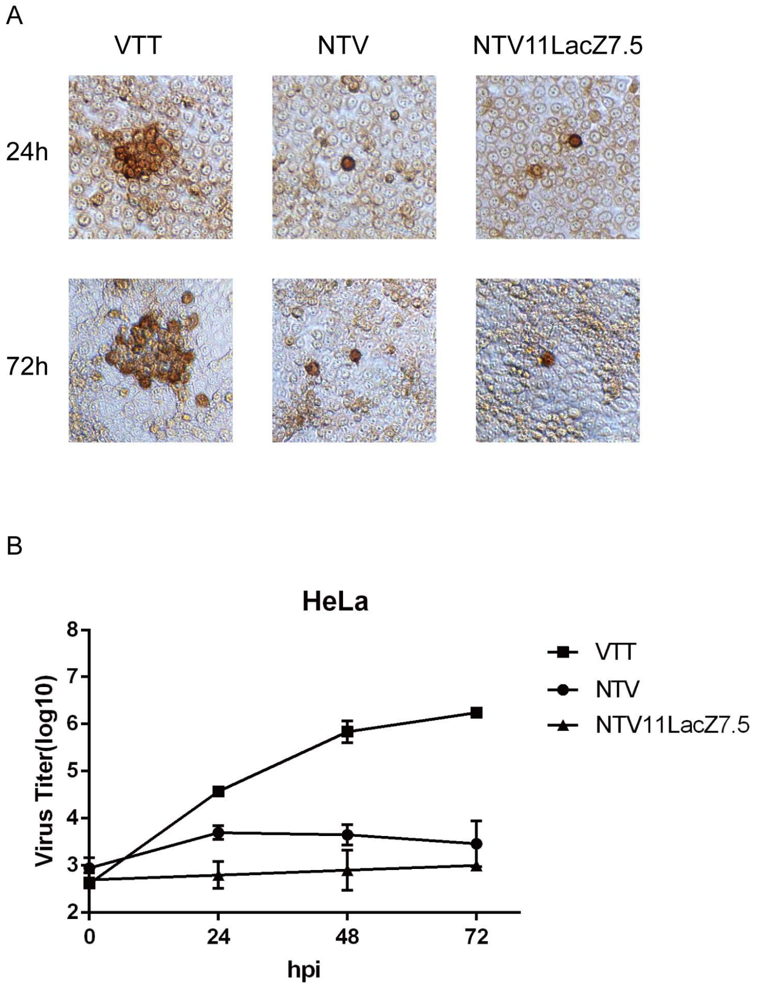

Supplementary Figure 2. Cell to cell dissemination and growth curves of NTV11LacZ7.5 in HeLa cells.

(A) HeLa cells were infected with VTT, NTV or NTV11LacZ7.5 at an MOI of 0.005, fixed at 24, or 72 hours p.i. and immunostained with anti-VACV antibody, and then visualized using DAB reagent kit. VTT was used as control. All images were taken at 200X magnification.

(B) HeLa cells were infected with VTT, NTV or NTV11LacZ7.5 at an MOI of 0.01, and harvested at 0, 12, 24, 48, and 72 hours p.i. and tittered by plaque assay in CEFs after freeze-thawing for three times. VTT was used as control. Data were acquired by three independent replicate experiments. Virus growth kinetic curves were graphed using Graphpad Prism 6.0.Colocalization of SAMD9 with G3BP1.

HeLa cells were mock infected or infected with VTT or NTV at
